# Adalimumab-Induced Pneumocystis jirovecii Pneumonitis (PCP) Misdiagnosed As Methotrexate-Induced Pneumonitis

**DOI:** 10.7759/cureus.88084

**Published:** 2025-07-16

**Authors:** Shayekh Ferdoush, Abir Aijaz, Shovan Rahman, Mustain Jawad, Muhammad Hamid

**Affiliations:** 1 Respiratory Medicine, University Hospitals Bristol and Weston, Weston-super-Mare, GBR; 2 Internal Medicine, University Hospitals Bristol and Weston, Weston-super-Mare, GBR; 3 Acute Medicine, University Hospitals Bristol and Weston, Weston-super-Mare, GBR; 4 Internal Medicine, Weston General Hospital, Weston-super-Mare, GBR

**Keywords:** adalimumab (humira), drug-induced pneumonitis, pneumocystis carinii pneumonia, pneumocystis jiroveci pneumonia, rheumatoid lung disease

## Abstract

*Pneumocystis jirovecii* pneumonia (PCP), a life-threatening opportunistic infection, occasionally mimics drug-induced pneumonitis, posing a diagnostic challenge in immunocompromised patients (such as those with rheumatoid arthritis, RA, on immunosuppressive therapy). We present a 46-year-old woman with seropositive RA treated with long-term methotrexate and recently initiated on adalimumab, who presented with progressive dyspnea, dry cough, and low-grade fever. Imaging revealed bilateral interstitial and ground-glass opacities, initially attributed to methotrexate-induced pneumonitis (MTX-IP). Methotrexate was discontinued, and corticosteroids were started, but clinical deterioration ensued. Bronchoalveolar lavage with polymerase chain reaction (PCR) confirmed PCP, leading to a revised diagnosis. The patient responded well to trimethoprim-sulfamethoxazole and corticosteroids, with full recovery and resolution of pulmonary findings. This case highlights the importance of distinguishing PCP from drug-induced lung toxicity, particularly in patients receiving tumor necrosis factor-α inhibitors like adalimumab, which significantly increase susceptibility to opportunistic infections. MTX-IP usually occurs within the first year, whereas PCP may develop insidiously. Elevated lactate dehydrogenase levels and poor response to corticosteroids should prompt further investigation. Early use of diagnostic tools such as bronchoalveolar lavage with PCR is critical for timely diagnosis and treatment. Clinicians must maintain a high index of suspicion for PCP in RA patients with new pulmonary symptoms while on biologic therapy.

## Introduction

*Pneumocystis jirovecii* pneumonia (PCP), a potentially life-threatening opportunistic infection, predominantly affects immunocompromised individuals, including those with HIV/AIDS, malignancies, organ transplants, and autoimmune diseases receiving immunosuppressive therapies [1-3]. Of late, the incidence of PCP has notably increased among non-HIV immunocompromised populations, particularly patients with other diseases like rheumatoid arthritis (RA) undergoing treatment with disease-modifying antirheumatic drugs and biologic agents [4,5].

Methotrexate, which is a cornerstone in RA management, has been associated with pulmonary toxicity, including methotrexate-induced pneumonitis (MTX-IP), a potentially rare but serious adverse effect [6]. Biologic agents like tumor necrosis factor (TNF) inhibitors (including adalimumab) have been implicated in increasing the susceptibility to opportunistic infections like PCP, although the incidence remains low [7]. The clinical manifestations of PCP, including nonproductive cough, dyspnea, and hypoxia, overlap with those of drug-induced pneumonitis, complicating the diagnostic process and potentially delaying targeted treatment [8,9].

Distinguishing between MTX-IP and PCP is critically important due to divergent management strategies, with the former necessitating immunosuppressive cessation and corticosteroid therapy. In contrast, the latter requires antimicrobial treatment with trimethoprim-sulfamethoxazole and possible adjunctive corticosteroids [10]. Radiologically, both these entities are characterized by bilateral ground-glass opacities on high-resolution computed tomography, further challenging diagnostic accuracy [11-13].

This report presents a diagnostically challenging case of a patient with RA treated with methotrexate and adalimumab who was initially misdiagnosed with MTX-IP but was ultimately found to have PCP. This case highlights the importance of considering opportunistic infections in the differential diagnosis of pulmonary symptoms in immunosuppressed patients and underscores the need for heightened clinical suspicion and early diagnostic evaluation.

## Case presentation

A 46-year-old woman with a history of RA, diagnosed in 2020, was admitted to the hospital on April 14, 2025, with complaints of shortness of breath, dry cough, and a single episode of fever lasting for two weeks. The patient had been on methotrexate (20 mg, recently increased to 25 mg three weeks prior) and adalimumab (started 10 weeks earlier) for her RA. Her rheumatological symptoms had been stable before the onset of these respiratory complaints.

**Figure 1:**
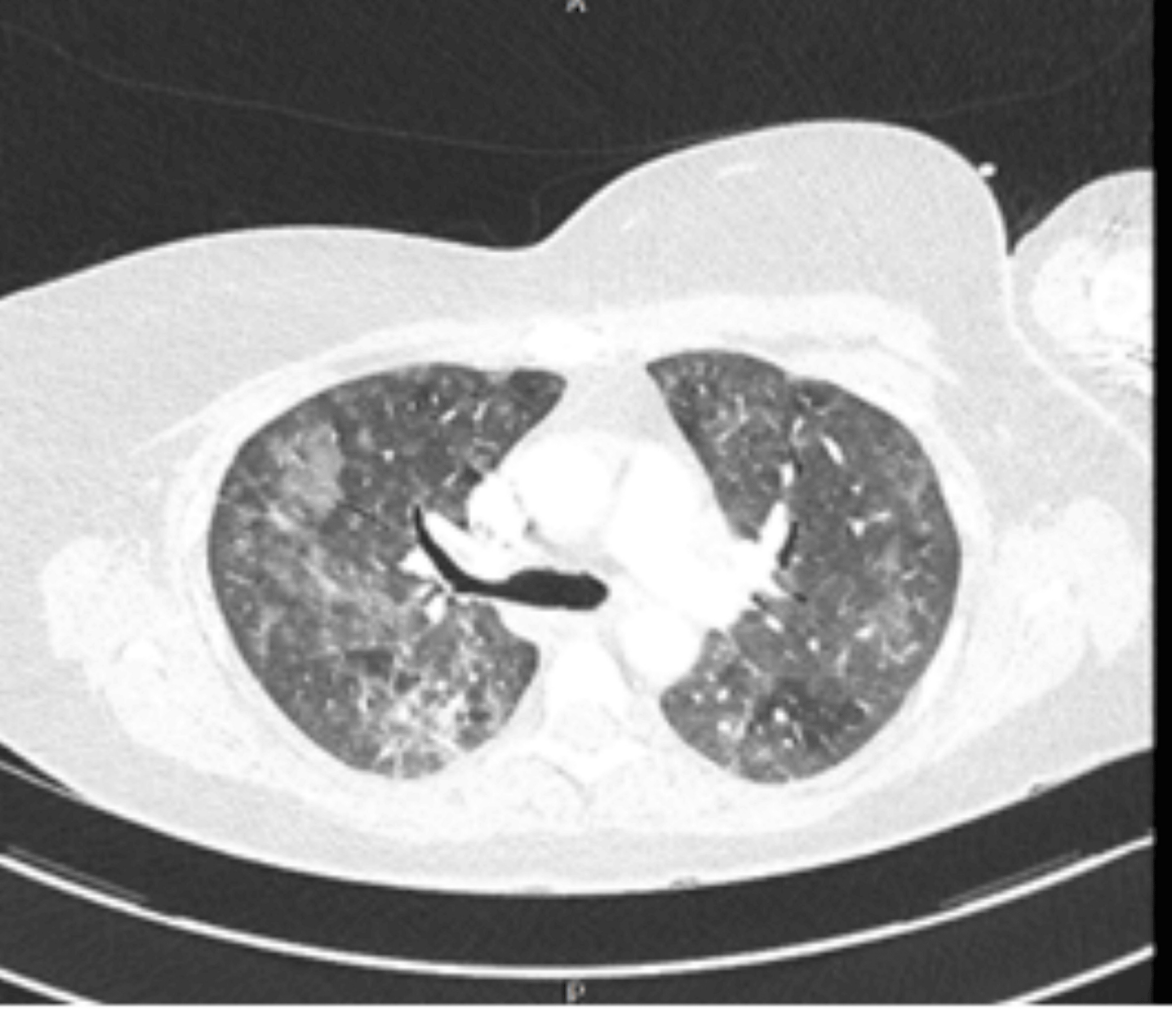
Computed tomography scan of chest revealing bilateral infiltrates

**Figure 2:**
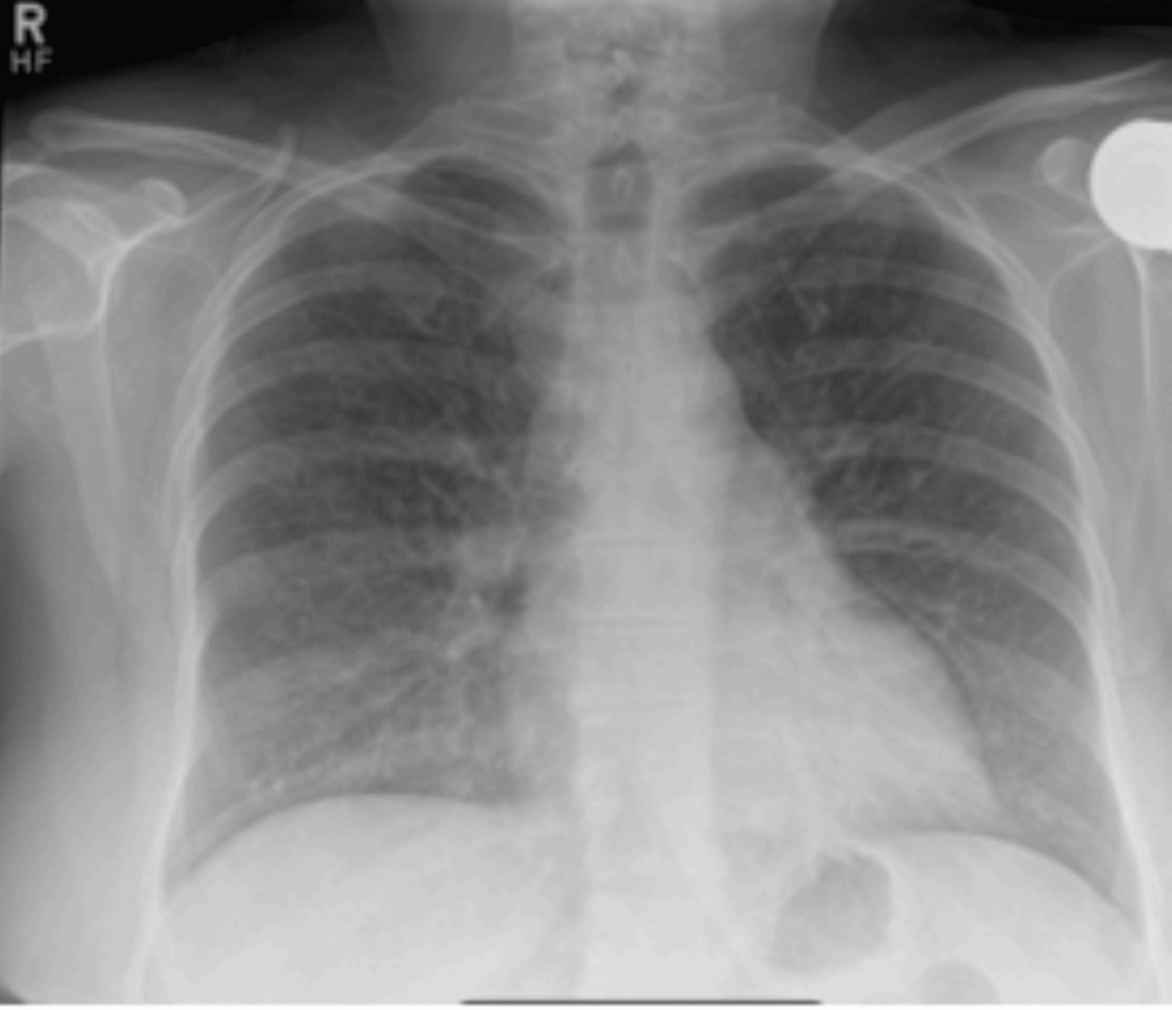
Bilateral infiltrates noted on the chest X-ray

The patient was a nonsmoker with no known exposure to asbestos, silica, or molds. Upon admission, she required 4 L of oxygen. Her clinical examination revealed bilateral crepitations, primarily in the mid-zones of the lungs on chest auscultation. Blood investigations revealed neutrophilia with high beta-d-glucan levels (Table 1). CT chest of this patient revealed diffuse bilateral alveolar infiltrates with ground-glass opacities without distortion of the pulmonary parenchyma. These findings were consistent with MTX-IP (Figures 1, 2).

**Table 1:** Investigations of the patient CRP: C-reactive protein; WBC: white blood cell

Investigations	Findings	Normal values
Neutrophils	6.46 × 10^9^/L	1.5-8 × 10^9^/L
Beta-D-glucan	206 pg/mL	<60 pg/mL
Procalcitonin	0.7 ng/mL (not indicative of a bacterial infection)	<0.1 ng/mL
CRP	176 mg/dL	<1 mg/dL
WBC	9 × 10^9^/L	4-11 × 10^9^/L

The patient was initially started on high-dose corticosteroids, assuming MTX-IP, and was discussed at the lung multidisciplinary team for further management. However, due to elevated beta-D-glucan levels and a positive polymerase chain reaction (PCR) for *P. jirovecii*, which were strongly suggestive of PCP, the management was adjusted.

Antibiotic therapy with co-trimoxazole 2 g four times a day IV was initiated for suspected PCP, and Tazocin was administered for seven days to cover any possible bacterial infection. By April 22, the patient's oxygen requirement had decreased, and she was no longer requiring respiratory support. Her clinical symptoms had significantly improved, confirming the diagnosis of PCP. Proper consent has been taken from the patient.

## Discussion

MTX-IP is a well-documented but relatively uncommon complication associated with methotrexate therapy. It typically presents with nonspecific respiratory symptoms, including cough, fever, and dyspnea, and is supported radiographically by findings such as bilateral ground-glass opacities. These features can closely mimic those of PCP, a serious opportunistic infection seen in immunocompromised patients. In the clinical case under discussion, the initial diagnosis of MTX-IP was based on symptomatology and imaging results, consistent with previously reported cases in the literature [6].

However, the overlapping clinical and radiological features between MTX-IP and PCP pose a significant diagnostic challenge. PCP is caused by *P. jirovecii*, a fungal organism that frequently affects patients undergoing immunosuppressive therapy. While methotrexate itself has immunosuppressive properties, the concurrent use of biologic agents such as adalimumab, a TNF-α inhibitor, significantly elevates the risk of opportunistic infections, including PCP [14]. In the present case, the presence of elevated serum beta-D-glucan levels, a biomarker associated with fungal infections, prompted clinicians to reconsider the initial diagnosis and evaluate the possibility of PCP. Beta-D-glucan has been shown in multiple studies to have high sensitivity (up to 90%) for diagnosing PCP in non-HIV immunocompromised patients. However, its specificity may vary depending on coexisting fungal infections [15].

Evidence from observational studies further supports the need for caution when diagnosing MTX-IP. For instance, a retrospective cohort study by Park et al. analyzing 50 patients with presumed MTX-IP found that nearly 20% were eventually diagnosed with infectious causes, including PCP, after microbiological evaluation and treatment response were considered. This diagnostic uncertainty is echoed in other case series and reviews that emphasize the clinical and radiographic similarities between MTX-IP and PCP, making early differentiation difficult without invasive procedures such as bronchoscopy and PCR testing [16].

Following the initiation of co-trimoxazole therapy in this case, a marked clinical improvement was observed, confirming PCP as the underlying pathology. This therapeutic response underscores the necessity of distinguishing between MTX-IP and PCP early in the clinical course, as their management strategies diverge significantly. While MTX-IP typically requires immediate cessation of methotrexate and initiation of corticosteroids, PCP demands targeted antimicrobial treatment. Delay in initiating PCP-specific therapy has been associated with significantly worse outcomes, especially in non-HIV patients who tend to present with more acute respiratory failure and higher mortality rates [17].

This case highlights the diagnostic ambiguity that arises due to the overlapping clinical and imaging characteristics of MTX-IP and PCP. Several reports in the literature describe misdiagnosis in similar scenarios, underscoring the need for heightened clinical vigilance. Moreover, the use of biologic agents such as adalimumab necessitates a broader differential diagnosis in patients presenting with pulmonary symptoms. Clinicians should maintain a high index of suspicion for PCP in such cases, especially when serological markers such as beta-D-glucan are elevated or when there is a poor response to steroid therapy alone. Early consideration of PCP can prevent diagnostic delays and improve clinical outcomes.

Differentiating between MTX-IP and PCP remains a critical challenge in patients on immunosuppressive therapy. Given the significant overlap in clinical presentation and imaging findings, a multidisciplinary approach incorporating laboratory markers, imaging, and clinical judgment is essential. The diagnostic process should be guided not only by clinical suspicion but also by available biomarkers and therapeutic response. This case illustrates the potential consequences of diagnostic misclassification and emphasizes the need for awareness of opportunistic infections in immunocompromised individuals.

## Conclusions

This case highlights the diagnostic complexity encountered in immunocompromised patients presenting with respiratory symptoms, particularly those receiving immunosuppressive therapy such as methotrexate and biologic agents. The clinical and radiographic similarities between MTX-IP and PCP can obscure accurate diagnosis, leading to potential delays in appropriate treatment. In this context, maintaining a broad differential diagnosis is crucial, as misdiagnosis may result in the continuation of immunosuppressive therapy in a patient with an active opportunistic infection, or conversely, the unnecessary discontinuation of effective treatment for autoimmune disease.

The timely use of specific diagnostic tools, including serum beta-D-glucan levels, PCR assays, and bronchoalveolar lavage, can aid in distinguishing PCP from other causes of pneumonitis. Early identification of PCP is critical, as prompt initiation of antimicrobial therapy significantly improves clinical outcomes. This case underscores the importance of clinical vigilance and diagnostic precision when evaluating pulmonary complications in immunosuppressed individuals. Ultimately, a multidisciplinary approach involving rheumatology, infectious disease, and pulmonology specialists is recommended to optimize patient care and reduce the risk of morbidity and mortality associated with diagnostic delays in this vulnerable population.
